# Depression during pregnancy and associated factors among women in Ethiopia: a systematic review and meta-analysis

**DOI:** 10.1186/s12884-024-06409-y

**Published:** 2024-03-26

**Authors:** Solomon Shitu Ayen, Abebaw Wasie Kasahun, Amare Zewdie

**Affiliations:** 1 Department of Midwifery, College of Medicine and Health Science, Wolkite University, Wolkite, 07 Ethiopia; 2 Department of Public Health, College of Medicine and Health Science, Wolkite University, Wolkite, Ethiopia

**Keywords:** Depression during pregnancy, Pregnant women, Ethiopia

## Abstract

**Background:**

Pregnancy is one of the most remarkable experiences in a woman’s life. Prenatal depression, characterized by stress and worry associated with pregnancy, can reach severe levels. On a global scale, mental and addictive disorders affect more than one billion people, causing 19% of years lived with disability. It is estimated that 25–35% of pregnant women experience depressive symptoms, with 20% meeting the diagnostic criteria for major depression.

**Methods:**

A systematic review and meta-analysis were conducted to examine depression during pregnancy in Ethiopia. The search was conducted from March 1–31, 2023. Data extraction used Microsoft Excel, and analysis was performed using STATA version 17. The New Castle-Ottawa Scale quality assessment tool was employed to evaluate the methodological quality of included studies. The Cochrane Q test and I2 statistics were used to assess heterogeneity. A weighted inverse variance random-effects model estimated the pooled level of antenatal depression (APD). Publication bias was detected using a funnel plot and Begg’s and Egger’s tests.

**Results:**

Out of 350 studies searched, 18 were included in the analysis. The overall pooled prevalence of depression in Ethiopia was 27.85% (95% CI: 23.75–31.96). Harari region reported the highest prevalence (37.44%), while Amhara region had the lowest (23.10%). Factors significantly associated with depression included unplanned pregnancies, low social support, low income, previous history of depression, intimate partner violence, and history of abortion.

**Conclusion:**

This systematic review and meta-analysis demonstrate that approximately one-quarter of pregnant women in Ethiopia experience depression during pregnancy. Unplanned pregnancy, low social support, low income, previous history of depression, history of abortion, and intimate partner violence are determinants of depression. To address this high prevalence, the Ethiopian government and stakeholders should develop policies that incorporate counseling during pregnancy follow-ups. Improving the quality of life for pregnant women is crucial for the well-being of families, communities, and the nation as a whole.

**Supplementary Information:**

The online version contains supplementary material available at 10.1186/s12884-024-06409-y.

## Introduction

Pregnancy is a significant life event for women, involving numerous physical, emotional, and social changes. This period can heighten the susceptibility of women and their fetuses to mental, physical, and psychological health issues. Among various mental health problems that occur during pregnancy, depression is the most prevalent psychiatric disorder affecting pregnant women. Depression during pregnancy, also known as antenatal depression (APD), is caused by anxiety and stress related to pregnancy and can stem from hormonal fluctuations, psychological disturbances, relationship issues, family or personal history of depression, life stress, low family support, unintended or unplanned pregnancies, and violence. If left untreated, APD can lead to adverse pregnancy outcomes, such as hypertension, low birth weight babies, preterm birth, and postnatal depression.

Depression is characterized by symptoms like excessive sleeping or insomnia, difficulty concentrating, forgetfulness, emotional instability, extreme irritability, fatigue, overeating or appetite loss, decreased interest in sex, feelings of guilt, sadness, suicidal thoughts, or death ideation. Globally, over one billion people suffer from mental or addictive disorders, accounting for 19% of all years lived with disability. Depression affects more than 350 million people across all age groups, with women being more vulnerable than men. Studies suggest that 25–35% of pregnant women experience depressive symptoms, and 20% of them meet the diagnostic criteria for major depression. The prevalence of APD varies in different countries, ranging from 20% in the United States to 30% in Finland and 35–50% in some lower and middle-income countries (LMICs).

In Africa, mental health issues among pregnant women and mothers have been extensively reported. APD is a critical public health concern in developing nations due to its intergenerational impact on mothers, infants, and children. In Ethiopia, the prevalence of APD and its associated factors differ across regions and time. Factors like older age, less education, being single, unemployment, low income, intimate partner violence, lack of social support, unplanned pregnancies, previous pregnancy loss, history of abortion, obstetric complications, and substance abuse contribute to APD.

Previous studies have provided inconsistent findings regarding APD prevalence and predictors at the national level, leaving policymakers with inconclusive information. This systematic review and meta-analysis aim to estimate the pooled prevalence of depression during pregnancy and its predictors among pregnant women in Ethiopia. By including recent studies and a larger sample of research articles (18 in total), this study offers a more comprehensive understanding of APD in Ethiopia.

## Methods

### Study design and setting

 To ensure the originality and avoid redundancy of the research, the International Prospective Register of Systematic Reviews (PROSPERO) database was consulted. Upon checking the database (http://www.library.ucsf.edu/), no published or ongoing research related to antenatal depression (APD) in Ethiopia was found. To proceed with the research, the protocol of the systematic review and meta-analysis was registered in the PROSPERO database with the ID CRD42023423700. The study adheres to the Preferred Reporting Items for Systematic Review and Meta-Analysis (PRISMA) guidelines to maintain the highest standards of research transparency and quality [1].

### Data sources and search strategy

Articles included in the review were searched from the following databases: MEDLINE, Scopus, PubMed, Science Direct, Google Scholar, African Journals Online, and Web of Sciences to retrieve related articles. Search terms were formulated using PICO guidelines through online databases. Medical Subject Headings (MeSH) and key terms had been developed using different Boolean operators ‘AND’ and ‘OR’. Each database was searched from its start date to March 2023 by using the following words: Depression (Mesh), depress∗(all fields), “antepartum depression” (Mesh), “antenatal depression” (Mesh), Ethiopia(Mesh), “prenatal depression” (Mesh), “pregnancy and depression” (Mesh), mental health (all fields). Furthermore, librarians were consulted to find unpublished research works on our area of interest for this review. The literature search was conducted by two separate researchers (SSA and AWK) to avoid missing articles.

### Eligibility criteria

In this systematic review and meta-analysis, the scope was to include all community and facility-based studies that reported the prevalence of antepartum depression (APD) and its determinants. This comprehensive approach aimed to ensure the study captured as much relevant information as possible. Both published and unpublished studies were considered to minimize potential publication bias. If multiple reports of the same study existed, the most recent and comprehensive study was chosen to ensure the analysis was based on the latest and most accurate data available.

The language restriction for the included articles was limited to English to facilitate the understanding and analysis of the data. This decision was made considering the limitations of the research team’s language capabilities and the widespread use of English in academic and scientific publications.

The timeframe for the search and inclusion of studies was set until March 30, 2023. This date allowed for a relatively recent and up-to-date analysis of the prevalence of APD and its associated factors in Ethiopia. As new research continues to emerge, future updates to the review may consider extending the search period or re-evaluating the study to incorporate more recent findings.

### Exclusion criteria

To maintain the focus and accuracy of the systematic review and meta-analysis, several exclusion criteria were applied. Articles that did not report the outcome variables of interest, such as the prevalence of antepartum depression (APD) or its determinants were excluded. This ensured that only relevant data was considered in the analysis.

Additionally, certain study types were excluded to maintain the quality and reliability of the review. Systematic reviews, case series, commentaries, conference abstracts, letters to editors, technical reports, qualitative studies, and other opinion publications were not included, as they might not provide sufficient quantitative data or meet the required methodological standards.

To avoid double-counting studies, potential duplicates were also excluded. This involved removing studies conducted in the same area with similar findings during the same study period. This step ensured that the final analysis included a diverse and representative sample of studies, reducing the risk of overrepresentation or redundancy in the data.

### Measurement of the outcome of interest

In this systematic review and meta-analysis, the primary focus was on determining the prevalence of antepartum depression (APD) in Ethiopia. The pooled prevalence was calculated as the primary outcome variable to provide an overall estimate of the proportion of affected individuals within the studied population.

The secondary outcome variable was to identify the factors associated with APD. To achieve this, a pooled Adjusted Odds Ratio (AOR) with 95% Confidence Intervals (CIs) was employed. This statistical measure allowed for the estimation of the strength and significance of the relationship between various determinants and the occurrence of APD.

To assess depressive symptoms in the studies, participants were typically asked specific questions. If they responded affirmatively (“Yes”) to these questions, they were considered to be experiencing depression. On the other hand, if they responded negatively (“NO”), they were not classified as depressed. This approach enabled the identification and analysis of individuals with potential APD within the studied population.

### Data extraction strategy

To ensure a comprehensive and organized review process, several steps were taken to manage and analyze the collected data. Initially, duplicate articles were identified and removed using EndNote X8, citation management software. This helped to avoid any overlapping data or double-counting of studies in the analysis.

Data extraction was carried out using a Microsoft Excel spreadsheet, which was designed with a pre-settled and piloted format. The format was adapted from the Joanna Briggs Institute (JBI) data extraction format to ensure a standardized and thorough approach to data collection. The extracted information included:

The primary author’s name, publication year, study year, study design, study area, study setup, sample size, response rate, data collection technique, The proportion of APD, adjusted Odds Ratio (AOR) with their 95% confidence interval. To facilitate the data extraction process, multiple researchers (SS, AW, and AZ) worked separately, ensuring a more accurate and reliable data collection. They used 2 by-2 tables for the second objective of the review, which focused on identifying factors associated with APD.

Finally, the data analysis was performed using STATA software version 17. This statistical software allowed for the appropriate handling and interpretation of the collected data, contributing to a more robust and reliable systematic review and meta-analysis.

### Quality assessment

The Newcastle-Ottawa Scale (NOS) was employed to assess the quality of the included studies in this systematic review. The NOS is a widely recognized and validated tool that evaluates the quality of non-randomized studies, such as cohort and case-control studies, in a structured manner. It focuses on three main components. The principal component, which evaluates the methodological quality of each primary study and is graded with five stars. The comparability of each study, which is graded from two stars and assesses the similarity of study participants and other factors that may influence the results. The outcomes and statistical analysis of each original study, graded from three stars and examining the adequacy of the reported results and statistical methods. The NOS uses a scoring system with a maximum score of 9 points. The quality of each study was rated based on the following scoring algorithms. A score of 7 points or more was considered “good” quality. A score between 2 and 6 points was considered “fair” quality. A score of 1 point indicated a “poor” quality study [2].

To ensure the validity and reliability of the systematic review results, only primary studies with fair to good quality were included in the analysis. The quality assessment was conducted by two authors (AW and SS), who evaluated the methodological quality, sample selection, sample size, comparability, and the outcome and statistical analysis of each original study.

### Data processing and analysis

After importing the selected articles into a Microsoft Excel spreadsheet, the data was exported to STATA version 17 for statistical analysis. The choice of statistical methods depended on the nature of the data and the presence of heterogeneity between studies.

To estimate the pooled prevalence of APD, a weighted inverse variance random-effects model was used. This model takes into account the variability between studies and provides a more conservative estimate when heterogeneity is present. The presence of statistical heterogeneity across the included studies was determined using Higgins I2 statistics and the Cochran-Q test. The interpretation of I2 values was based on the following criteria. 75–100%: considerable heterogeneity, 50–90%: substantial heterogeneity, 30–60%: moderate heterogeneity, 0–40%: mild heterogeneity [3].

When studies showed significant heterogeneity, a random-effects model was used. In cases of homogeneous studies, a fixed-effects model was applied. To assess the possibility of publication bias, a Funnel plot and Eggers test were conducted. Publication bias was considered justified if the *p*-value was greater than 0.05. The results were presented in a forest plot format, which displays the pooled prevalence of APD with its 95% confidence interval (CI).

For the analysis of the adjusted odds ratio (AOR) from eligible studies, along with their 95% confidence intervals, the pooled AORs were computed using either a random or fixed-effect model, depending on the presence of heterogeneity. Finally, forest plots were used to visually represent the pooled estimates for APD and its determinants, along with their respective 95% confidence intervals, allowing for a comprehensive understanding of the relationship between these factors and APD.

## Result

### Characteristics of included studies

In total, 350 studies were initially identified from various search engines, including 341 from the specified data sets and 9 from other sources. After removing duplicates, 63 studies were excluded, leaving 287 studies. Among these, 201 studies were further excluded based on their titles and abstracts, not meeting the inclusion criteria.

**Fig. 1:**
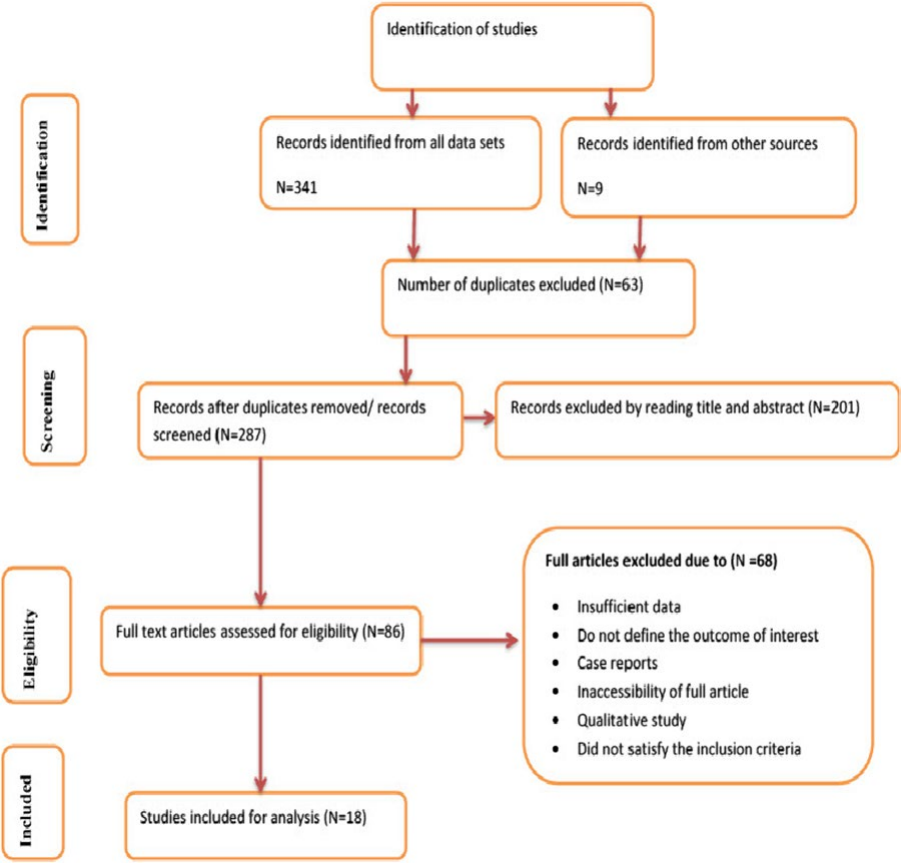
Flow chart of selection for systematic review and meta-analysis on DDP and associated factors in Ethiopia, 2023

Subsequently, 68 studies were excluded after reviewing their full texts due to insufficient data or not satisfying the predefined criteria. Finally, 18 studies were deemed eligible for inclusion in the analysis (Fig. 1). These studies were conducted across different regions of Ethiopia, with four studies each in the South Nations, Nationalities, and Peoples’ Region (SNNPR) and Oromia, three studies each in Amhara and Addis Ababa, two studies in Tigray, and one study in each of Somali and Harari (Table 1). The population exposure and outcome (PEO) of the study participants are demonstrated in Table 2, providing a clear overview of the study population and the outcomes assessed in the included studies.

**Table 1:** Study characteristics included in the systematic review and meta-analysis on the prevalence of DDP and its determinants in Ethiopia, 2023

Authors name	Study year	Study area	Design	Sample size	Response rate	Prevalence of APD	Study quality	Assessment tool
Birhane G. et al.	2020	Tigray	CS	203	100%	31.5%	Good	BDI
Dibaba et al. [4]	2013	Oromia	CS	627	99%	19.9%	Good	EPDS
Tilahun B. et al.	2017	Tigray	CS	196	93.8%	31.1%	Good	BDI
Edao T. et al.	2021	Somali	CS	403	98%	24.3%	Good	EPDS
Bitew et al. [5]	2016	SNNP	CS	1311	97%	29.5%	Good	PHQ-9
Ayele et al. [6]	2016	Amhara	CS	388	92.82%	23%	Good	BDI
Biratu and Haile [7]	2015	Addis Ababa	CS	393	93.13%	24.94%	Good	EPDS
Bisetegn et al. [8]	2016	Amhara	CS	527	97%	11.8%	Good	EPDS
Kasim et al.	2023	Addis Ababa	CS	415	98.6%	20.5%	Good	SRQ-20
Bekem et al.	2023	Oromia	CS	343	95%	27.6%	Good	EPDS
Tamiru et al. [9]	2022	Harari	CS	1,015	98.2%	37.5%	Good	SRQ-20
Tarafa et al. [10]	2022	Oromia	CS	406	96%	32.7%	Good	EPDS
Borie et al.	2022	SNNP	CS	313	98.7%	27.2%	Good	PHQ-9
Beketie et al. [11]	2021	SNNP	CS	323	97.8%	35.4%	Good	EPDS
Abebe et al. [12]	2022	Addis Ababa	CS	397	92.7%	47.6%	Good	PHQ-9
Beyene et al. [13]	2021	Amhara	CS	970	96.2%	27.76%	Good	EPDS
Shitu Ayen et al.	2020	SNNP	CS	343	96%	27.6%	Good	EPDS
Yonas and Liyew	2021	Oromia	CS	314	96.7%	16.6%	Good	PHQ-9

*SRQ* Self-Reporting Questionnaire, *EPDS *Edinburg Postnatal Depression Scale, *PHQ 9* Patient Health Questionnaire, *BDI* Beck Depression Inventory

**Table 2:** PEO table for systematic review and meta-analysis on the prevalence of DDP and its determinants in Ethiopia, 2023

Population	Exposure	Outcome
All reproductive-age pregnant women in Ethiopia	Being pregnant will expose the woman to depression	Either depressed or not depressed

### Sensitivity analysis

**Fig. 2:**
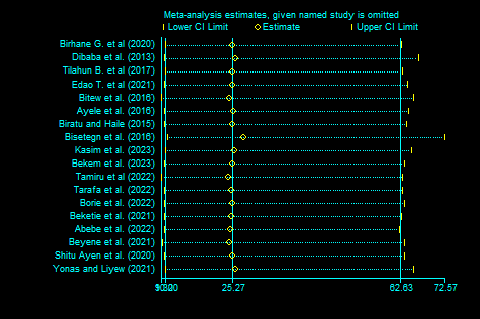
Sensitivity test of studies included in systematic review and meta-analysis on DDP and associated factors in Ethiopia, 2023

The sensitivity analysis, performed using the random-effects model, aimed to assess the influence of individual studies on the overall estimate of antepartum depression (APD) prevalence in Ethiopia. The results of this sensitivity analysis, presented in Fig. 2, indicate that no single study significantly impacted or unduly influenced the overall estimate of APD prevalence in Ethiopia. This finding suggests that the results are robust and stable, as the overall prevalence estimate remains consistent even when specific studies are removed from the analysis.

### Publication bias

**Fig. 3:**
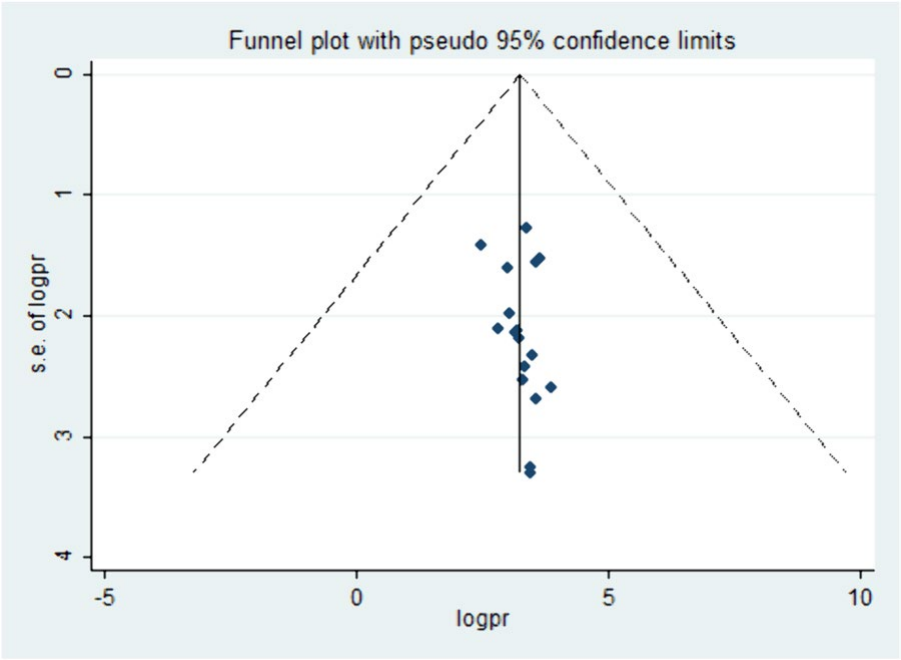
Funnel plot showing the symmetric distribution of articles on DDP in Ethiopia, 2023

To further evaluate the possibility of publication bias, funnel plots were employed to visually inspect the asymmetry of the distribution of study effect sizes against their standard errors. An inverted funnel shape that appears symmetrical suggests a low likelihood of publication bias. In this case, the funnel plot displayed a large, symmetrical inverted funnel shape (Fig. 3), indicating that the chance of publication bias is minimal.

In addition to the visual assessment, statistical methods such as Egger’s and Begg’s tests were used to provide supplementary evidence. A *p*-value less than 0.05 are generally considered statistically significant, and in this context, it suggests that there is no significant publication bias present in the included studies, supporting the visual assessment findings.

### The pooled prevalence of APD in Ethiopia

**Fig. 4:**
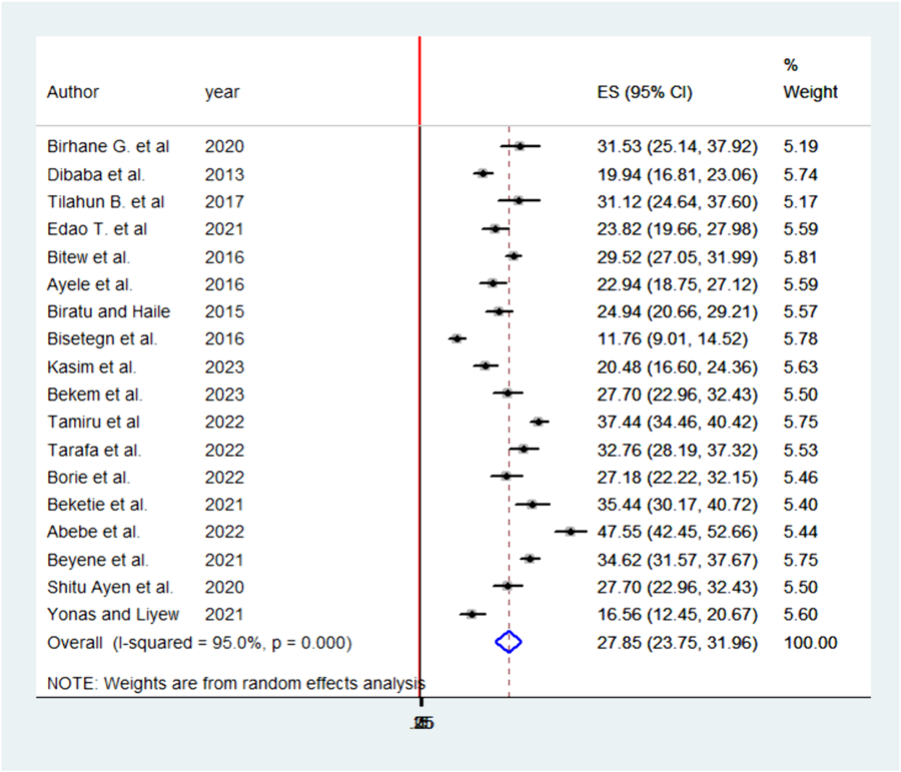
Pooled prevalence systematic review and meta-analysis on DDP and associated factors in Ethiopia, 2023

Antepartum depression in Ethiopia varied across the studies, demonstrating significant heterogeneity. The heterogeneity was quite high, with an I2 value of 95.0% and a statistically significant *P*-value less than 0.000. This high level of heterogeneity indicates that there are substantial differences in the study results that cannot be explained by chance alone. Despite this heterogeneity, the overall pooled prevalence of APD in Ethiopia was estimated to be 27.85% (95% confidence interval: 23.75–31.96). This combined prevalence figure is summarized using a forest plot (Fig. 4), which visually represents the individual study results and their contribution to the overall prevalence estimate.

The high heterogeneity among studies highlights the importance of considering the context and methodology of each individual study when interpreting the overall prevalence estimate. Further research and investigation may be required to understand the factors contributing to this variability and to develop targeted strategies for managing and preventing APD in Ethiopia.

### Subgroup analyses of APD in Ethiopia

**Fig. 5:**
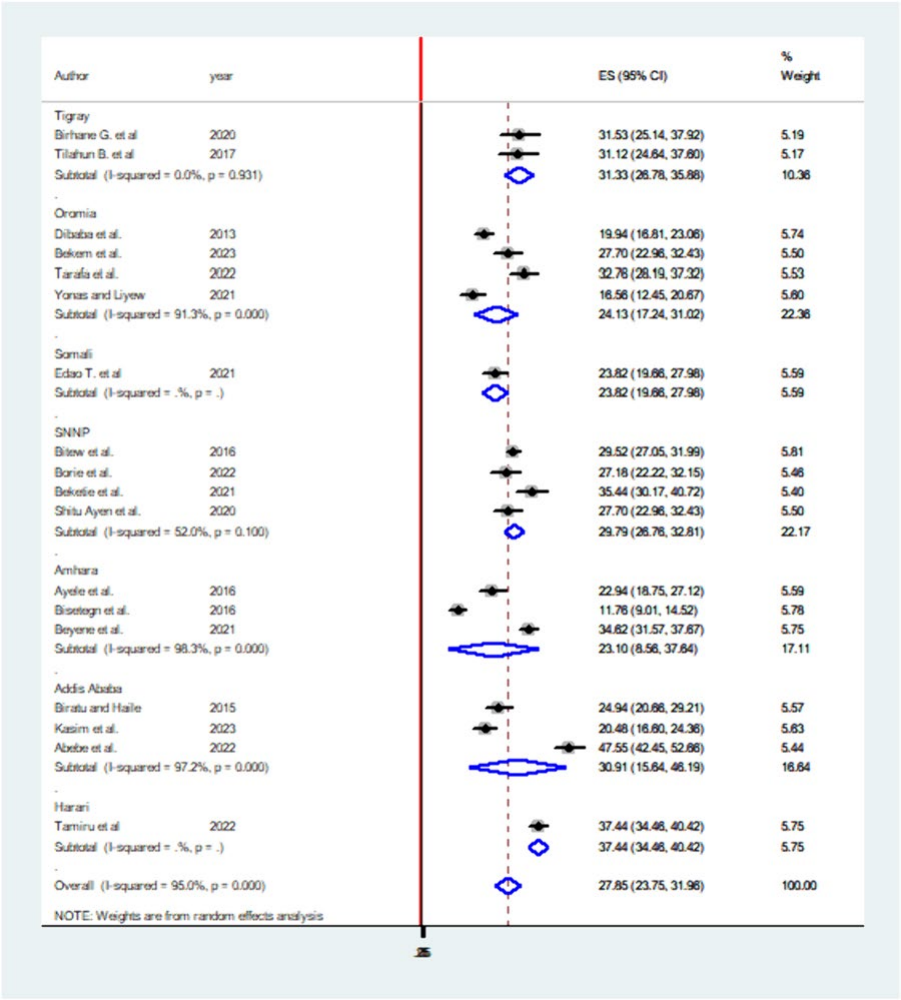
Subgroup analysis of systematic review and meta-analysis on DDP and associated factors in Ethiopia, 2023

Subgroup analyses were conducted by the study region. Accordingly, the highest prevalence of APD was reported in Harari and lowest in Amhara regions with prevalence and I^2^ of 37.44% (95% CI: 34.46–40.42) I^2^ 95% *P* = 0.000 and 23.10 (95% CI: 8.56–37.64) I^2^ 98.3% *P* = 0.000 respectively (Fig. 5).

### Factors associated with APD in Ethiopia

Six significant factors have been identified in this systematic review and meta-analysis to be associated with antepartum depression (APD) in Ethiopia. These factors are unplanned pregnancy, social support, history of abortion, intimate partner violence, low income, and previous history of depression. Each factor’s association with APD is quantified using adjusted odds ratios (AOR) and their respective confidence intervals.

Unplanned pregnancy: Women experiencing unplanned pregnancies have a 2.47 times higher likelihood of developing APD compared to those with planned pregnancies (AOR: 2.47, 95% CI: 1.92–3.19). Low social support and income: Pregnant mothers with low social support and income are 2 times more likely to develop APD than their counterparts (AOR: 2.06, 95% CI: 1.44–2.94 for low social support; AOR: 2.13, 95% CI: 1.54–2.91 for low income). Previous history of depression: Women with a history of depression have a 3 times higher likelihood of developing APD (AOR: 3.49, 95% CI: 2.40–5.08). History of intimate partner violence: Women with a history of intimate partner violence are 2.7 times more likely to develop APD than those without such history (AOR: 2.71, 95% CI: 2.07–3.55). History of abortion: Women with a history of abortion have a 2 times higher likelihood of developing APD compared to those without (AOR: 2.12, 95% CI: 1.392–3.25).

These findings, summarized in Table 3, provide valuable insights into the potential risk factors associated with APD in Ethiopia. Addressing these factors and implementing targeted interventions could help reduce the prevalence and impact of APD on affected women in the country.

**Table 3:** The pooled odds ratio of associated factors of a study/ systematic review and meta-analysis on the prevalence of DDP and its determinants in Ethiopia, 2023

Variables	Authors	AOR & CI in the previous studies	CI & AOR in this review
History of abortion	Bisetegn et al. Tamiru et al.	2.57 (95% CI: 1-6.61)	2.12 (95% CI: 1.392–3.25).
		2.03 (95% CI: 1.27–3.24)	
Intimate partner violence	Dibaba et al.	3.41 (95% CI: 1.18–9.1)	2.71 (95% CI: 2.07–3.55)
	Tamiru et al.	2.67 (95% CI: 2.02–3.53)	
Low income	Tilahun B. et al.	3.66 (95% CI: 1.12–11.96)	2.13 (95% CI: 1.54–2.91)
	Tarafa et al.	2.01 (95% CI: 1.29–3.14)	
	Abebe et al.	2.1 (95% CI: 1.31–3.36)	
Low social support	Edao T. et al.	3.34 (95% CI: 1.5–7.43)	2.06 (95% CI: 1.44–2.94)
	Biratu & Haile	1.89 (95% CI: 1.06 3.36)	
	Tarafa et al.	1.79 (95% CI: 1.14–3.37)	
Unplanned pregnancy	Dibaba et al.	1.96 (95% CI: 1.04–3.69)	2.47 (95% CI: 1.92–3.19)
	Biratu & Haile	2.78 (95% CI: 1.59–4.85)	
	Bisetegn et al.	2.39 (95% CI: 1.2–4.76)	
	Tarafa et al.	2.77 (95% CI: 1.71–4.54)	
	Beketie et al.	2.71 (95% CI: 1.21–6.07)	
	Shitu Ayen et al.	2.11 (95% CI: 1.05–4.44)	
Previous history of depression	Edao T. et al.	6.02 (95% CI: 2.31–10.01)	3.49 (95% CI: 2.40–5.08)
	Biratu & Haile	2.57 (95% CI: 1.48–4.48)	
	Bisetegn et al.	3.48 (95% CI: 1.71–7.06)	

## Discussion

This systemic review and meta-analysis provide valuable insights into the prevalence and associated factors of antepartum depression (APD) in Ethiopia. The pooled prevalence of APD in Ethiopia is 27.85% (95% CI: 23.75–31.96), which is in line with studies conducted in Nigeria, Ghana, and Africa as a whole [14–16], but higher than studies in Brazil, Nepal, South Africa, and rural Ghana [17–23]. The prevalence in Ethiopia might be higher due to differences in study periods, populations, and areas. However, the prevalence of this systemic review and meta-analysis was lower than in studies done in Tanzania, Côte d’Ivoire, Kenya [15, 24, 25] the reason might be due to most of the studies are single area findings which may not be strong too as compared to this systemic review which is pooled prevalence. Also, it may be due to the study period, study population, and study area differences.

Sub-group analysis reveals the highest prevalence in the Harari region (37.44%) and the lowest in the Amhara region (23.1%). Factors significantly associated with APD include unplanned pregnancy, social support, history of abortion, intimate partner violence, low income, and previous history of depression. These associations are quantified using adjusted odds ratios (AOR) and their respective confidence intervals.

Low social support and low income are associated with a twofold increased likelihood of developing APD. This finding is consistent with studies conducted in Nepal, Tanzania, Nigeria, South Africa, and systemic reviews in Africa. Women with unplanned pregnancies have a 2.47 times higher likelihood of developing APD, possibly due to economic stress and concerns related to unplanned pregnancies in developing countries [18–22, 24].

In this systemic review and meta-analysis, women with low income were two times more likely to develop APD than women with good income AOR 2.13; 95% CI; 1.54–2.91. The finding was in line with studies conducted in Tanzania, Nigeria, and systemic reviews in Africa [16, 21, 24]. This may be due to a family with poor income may be related to the economic problem and she became stressed thinking of how can she give care for the newborn and the family.

Women with a history of unplanned pregnancy were 2.47 times more likely to develop APD than women with planned pregnancy AOR 2.47; 95% CI; 1.92–3.19. The reason may be in developing countries planning to get pregnant will be associated with the economy of the household so women with unplanned pregnancies may be stressed by thinking of economic problems after birth which may lead to depressive conditions [21].

A previous history of depression has been associated with APD in this review. Women with a previous history of depression had 3.5 times more likely to develop APD than their counterparts AOR 3.49; 95% CI; 2.40–5.08. The finding was consistent with studies done in Brazil and South Africa [17, 20]. A possible explanation might be scientifically the reoccurrence of APD is most likely in consecutive pregnancies unless possible risk factors are avoided [20].

The probability of the women developing APD was 2.71 times more likely with intimate partner violence as compared to their counterparts AOR 2.71; 95% CI; 2.07–3.55. The finding was supported by studies in Kenya, Nigeria, and South Africa. The possible explanation might be as the name indicates violence is violating the rights of the person physically, sexually, or emotionally. Thus, women with violence might be more likely to develop depression. It may also be a partner who violates the rights of the woman may have poor educational conditions and can’t respect others [14, 19, 25].

Women with a history of abortion were two times more likely to develop depression during pregnancy than their counterparts AOR 2.12; 95% CI; 1.39–3.25. The possible reason might be women who experience abortion may be stressed due to fear of repeated abortion and loss of their baby. The finding was supported by a study done in Nigeria [21].

These findings can help guide interventions and policies to address the factors associated with APD in Ethiopia, ultimately reducing its prevalence and impact on affected women.

### Strengths and limitations of the study

The study presents several strengths and limitations. One of the main strengths lies in the comprehensive search strategy employed to retrieve related articles. By searching different databases and following the PRISMA flow charts strictly, the study ensures a systematic and thorough approach to identifying relevant studies. Additionally, including studies published over different years allows for an analysis of potential trends in antepartum depression (APD) during pregnancy in Ethiopia.

However, there are some limitations to consider. Firstly, the study is limited to English-language articles, which might have resulted in excluding relevant studies published in other languages. This could potentially affect the generalizability and comprehensiveness of the findings. Secondly, the absence of studies from some regions of Ethiopia might raise questions about the generalizability of the results across the entire country. This could be due to various factors, such as regional differences in access to healthcare, cultural practices, or research focus. To address these limitations, future studies could consider expanding their search to include non-English articles and aim to include studies from all regions of Ethiopia to enhance the generalizability of their findings.

## Conclusion

The findings of this systemic review and meta-analysis highlight the significant prevalence of antepartum depression (APD) in Ethiopia, affecting one-quarter of pregnant women. Addressing the determinant factors of depression, such as unplanned pregnancies, low social support, low income, previous history of depression, history of abortion, and intimate partner violence, is crucial for reducing the prevalence of APD.

To address this issue, the government of Ethiopia and other stakeholders should consider incorporating depression counseling during antenatal care (ANC) follow-ups. By providing information and support to pregnant women, they can better understand and cope with potential risk factors and seek appropriate help when needed. This approach not only improves the well-being of individual women but also contributes to the overall well-being of their families, communities, and the nation.

Incorporating depression awareness and prevention measures into ANC services can help create a supportive environment for pregnant women, empowering them to make informed decisions and seek assistance when facing challenges. This holistic approach to maternal health can lead to better mental health outcomes for pregnant women, ultimately benefiting the entire society.

### Supplementary Information

**Supplementary Material 1.**

**Supplementary Material 2.**

## Data Availability

The result of this systematic review and meta-analysis was extracted from the data gathered and analyzed based on the stated methods and materials. All the relevant data are within the paper.

## Abbreviations

AOR: Adjusted Odds Ratio
APD: Ante Partum Depression
DDP: Depression during Pregnancy
JBI: Joanna Briggs Institute
LMIC: Lower and Middle-Income Countries
NOS: Newcastle-Ottawa Scale
PRISMA: Preferred Reporting Items for Systematic Reviews and Meta-Analyses
SNNPR: South Nations, Nationalities, and People Region
WHO: World Health Organization
