# Correction: Depression during pregnancy and associated factors among women in Ethiopia: a systematic review and metaanalysis

**DOI:** 10.1186/s12884-024-06506-y

**Published:** 2024-04-23

**Authors:** Solomon Shitu Ayen, Abebaw Wasie Kasahun, Amare Zewdie

**Affiliations:** 1 Department of Midwifery, College of Medicine and Health Science, Wolkite University, Wolkite 07, Ethiopia; 2 Department of Public Health, College of Medicine and Health Science, Wolkite University, Wolkite, Ethiopia

**Correction: BMC Pregnancy Childbirth 24, 220 (2024)**

**https://doi.org/10.1186/s12884-024-06409-y**

Following the publication of the original article [1], it was noted that due to a typesetting error the figure legends were paired incorrectly. The figure legends for Figs. 2, 4 and 5 were wrongly given as captions for Figs. 5, 2 and 4 respectively.

The correct figures and captions have been included in this correction.

Also, there are some type errors in the main text submitted which need some modifications. Incorrect and correct data are indicate in bold.

In “*Data sources and search strategy*” section, there is the repetition of phrases:

“**antepartum depression” (Mesh), “antepartum depression” (Mesh)**” should be changed to “**antepartum depression” (Mesh)**”.

In “*The pooled prevalence of APD in Ethiopia*” section:

“**The prevalence of atrial fibrillation and flutter (APF)** in Ethiopia” should be changed to “**Antepartum depression** in Ethiopia”

“Despite this heterogeneity, the overall pooled prevalence of **APF** in Ethiopia” should be changed to “Despite this heterogeneity, the overall pooled prevalence of **APD** in Ethiopia”

In the last line, “and preventing **APF** in Ethiopia” should be changed to “and preventing **APD** in Ethiopia”.

In the “*Sensitivity analysis*” section:

“the overall estimate of **atrial fibrillation and flutter (APF)** prevalence in Ethiopia.” should be corrected as “the overall estimate of **antepartum depression (APD)** prevalence in Ethiopia.”

In the “Factors Associated with APD in Ethiopia” section:

“to be associated with **atrial premature depolarization (APD)** in Ethiopia.” should be corrected to “to be associated with **antepartum depression(APD)** in Ethiopia.”

The original article has been corrected.

**Fig. 1:**
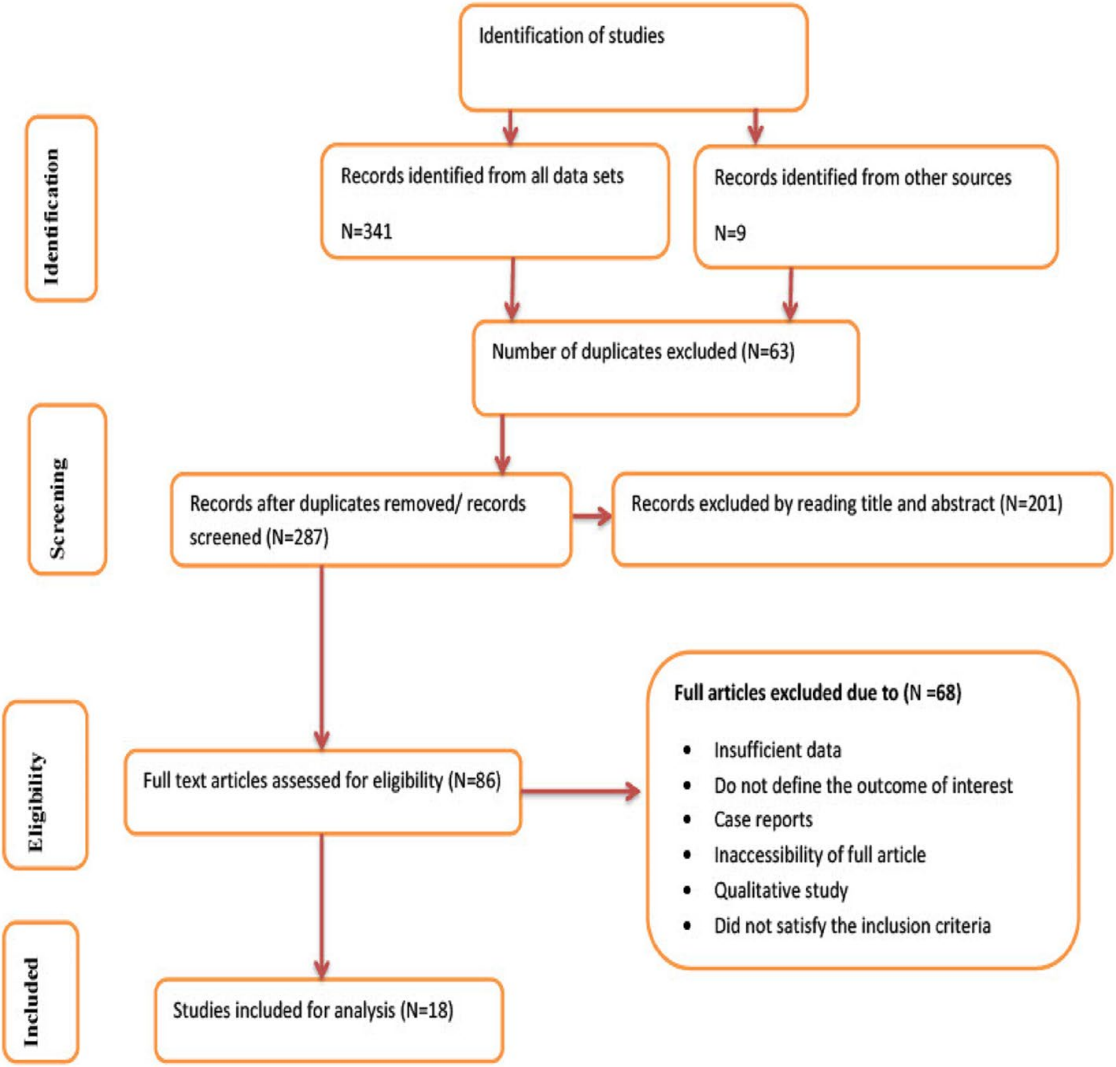
Flow chart of selection for systematic review and meta-analysis on DDP and associated factors in Ethiopia, 2023

**Fig. 2:**
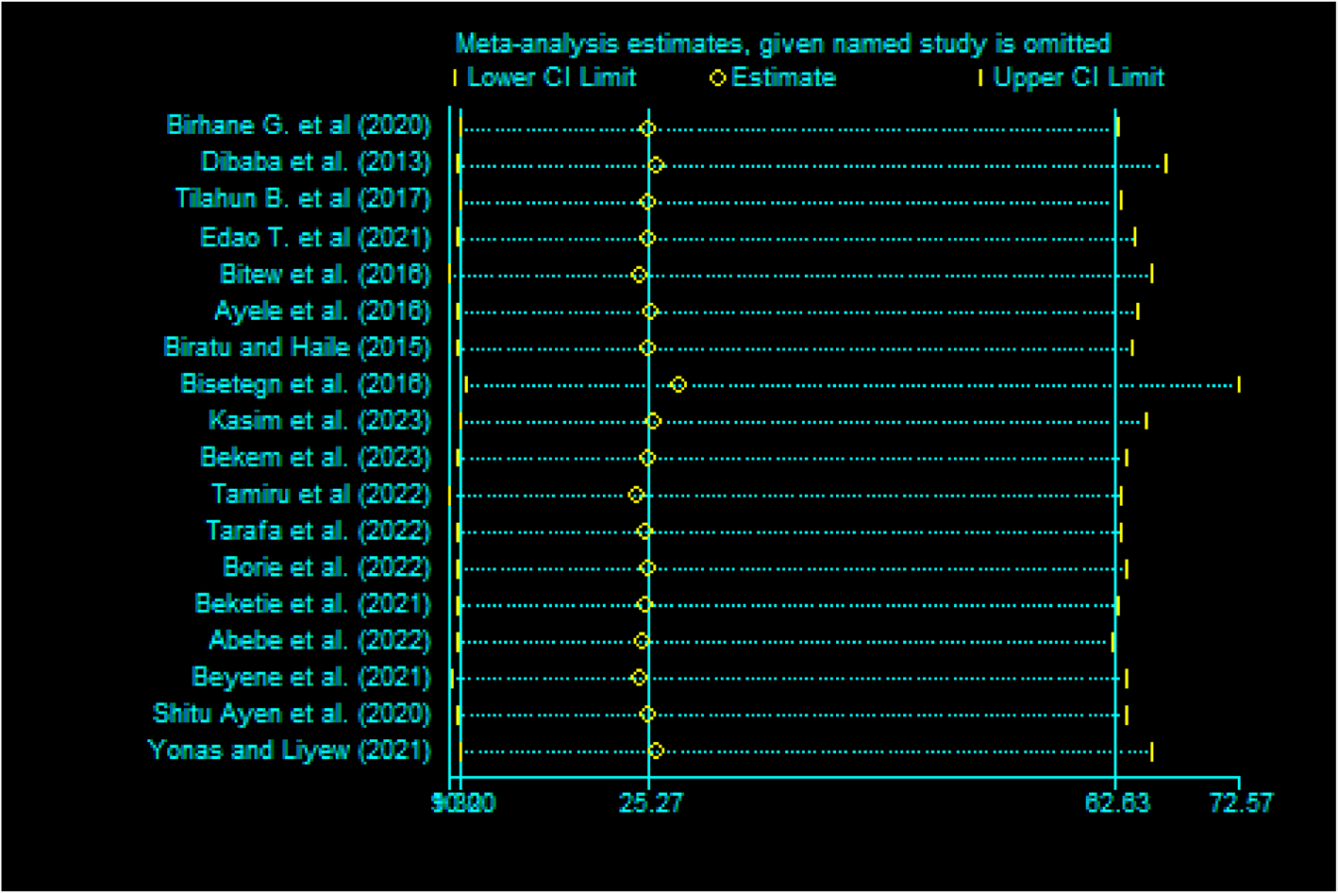
Sensitivity test of studies included in systematic review and meta-analysis on DDP and associated factors in Ethiopia, 2023

**Fig. 3:**
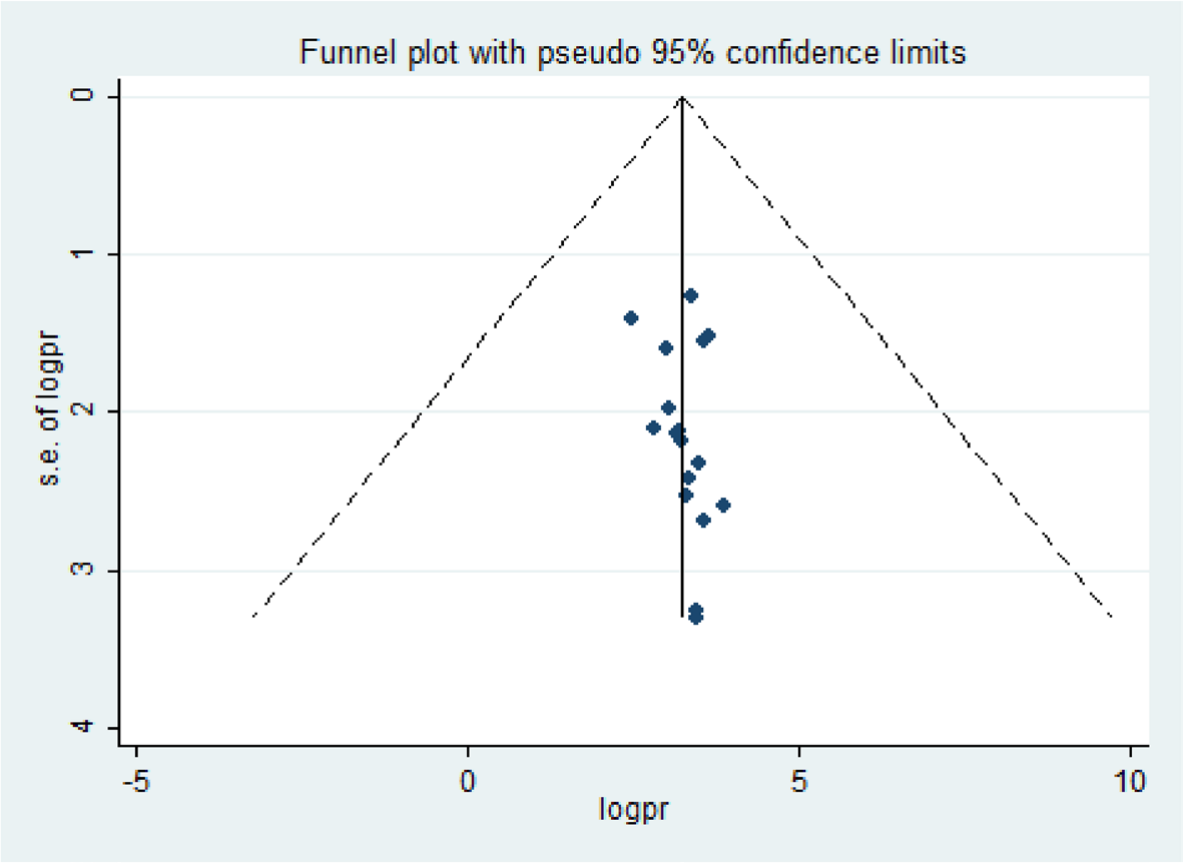
Funnel plot showing the symmetric distribution of articles on DDP in Ethiopia, 2023

**Fig. 4:**
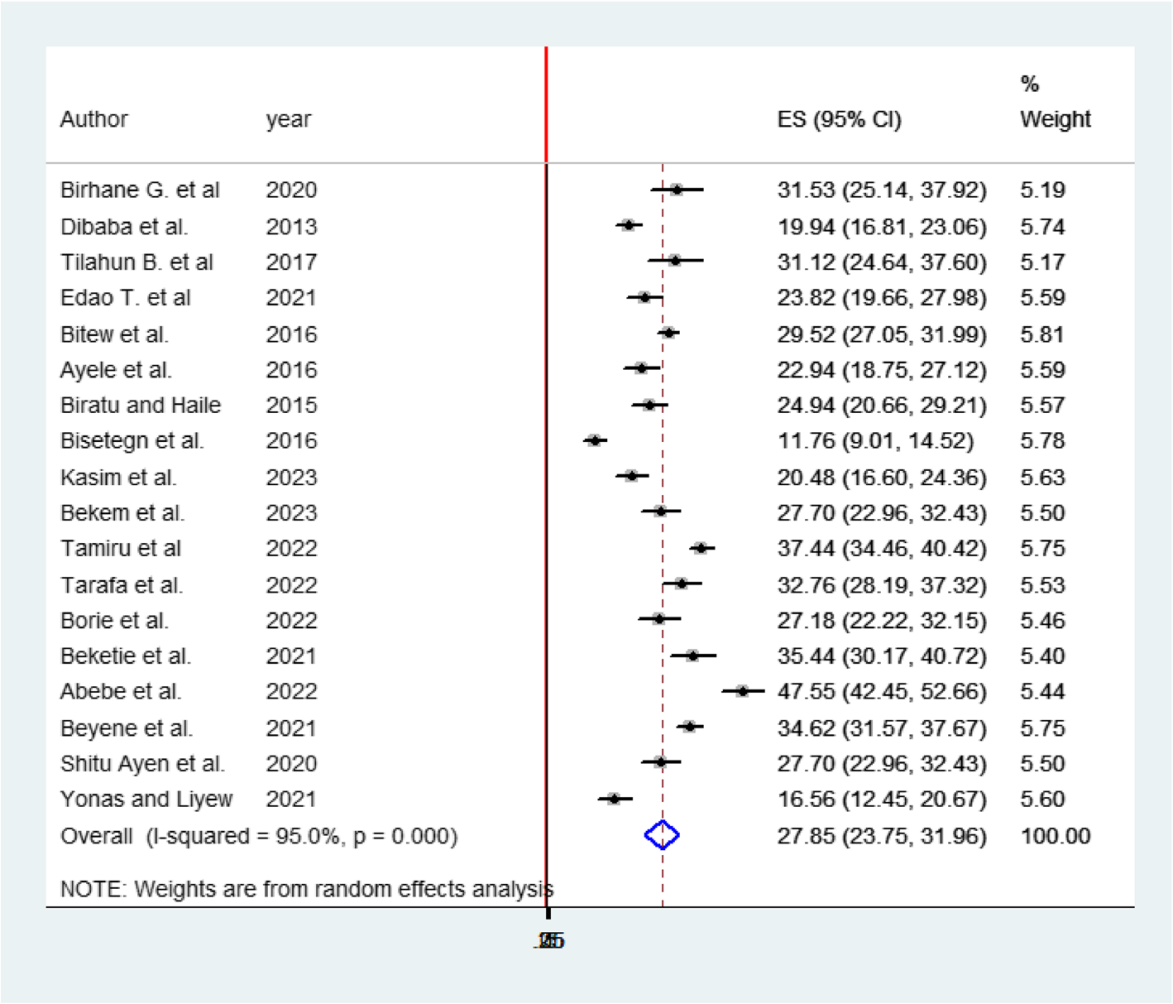
Pooled prevalence systematic review and meta-analysis on DDP and associated factors in Ethiopia, 2023

**Fig. 5:**
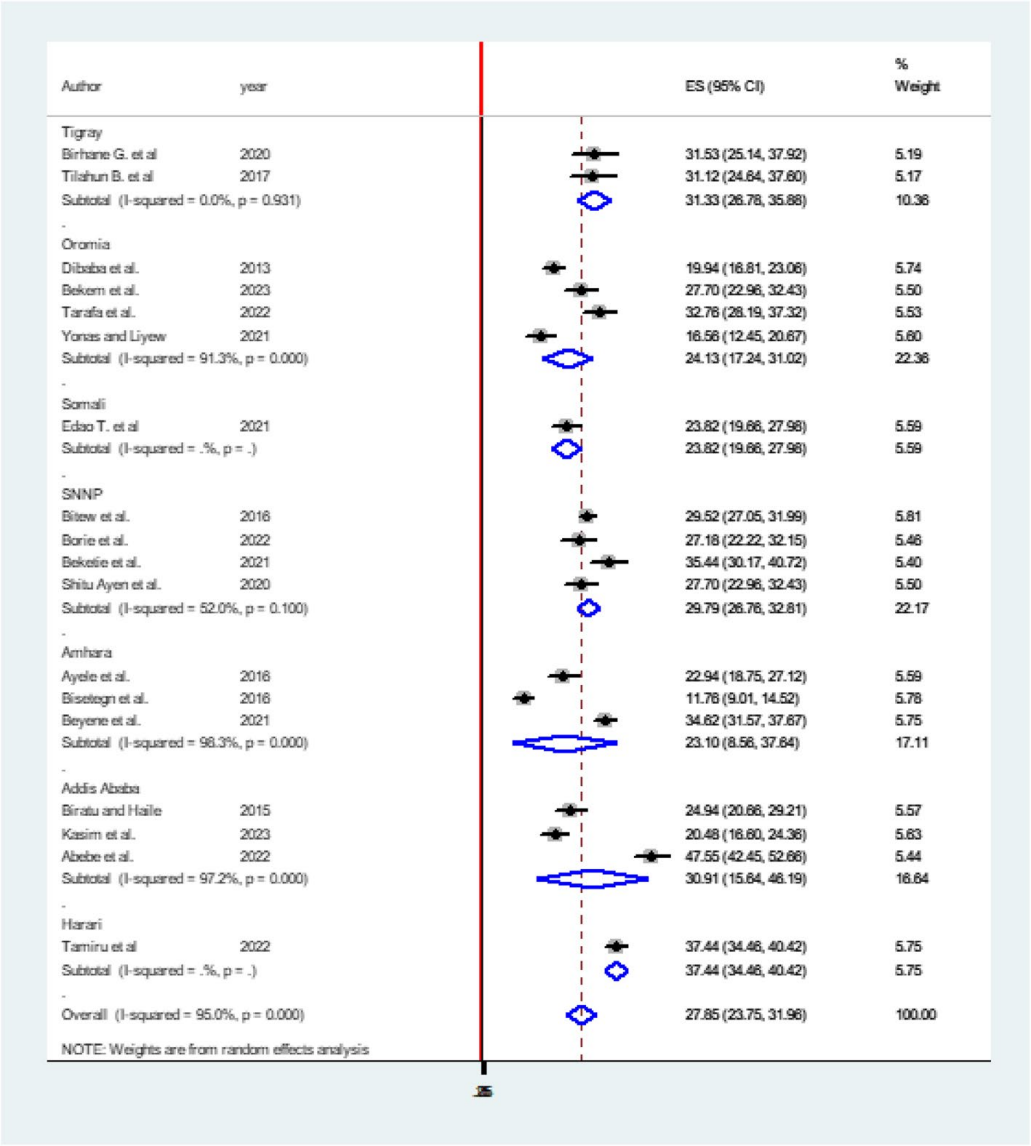
Subgroup analysis of systematic review and meta-analysis on DDP and associated factors in Ethiopia, 2023

## References

[1] Ayen SS, Kasahun AW, Zewdie A (2024). Depression during pregnancy and associated factors among women in Ethiopia: a systematic review and meta-analysis. BMC Pregnancy Childbirth.

