# Supplementary material for: Depression during pregnancy and associated factors among women in Ethiopia: a systematic review and meta-analysis
Source: BMC Pregnancy Childbirth. 2024 Mar 26;24:220. doi: 10.1186/s12884-024-06409-y (PMC10964661; doi:10.1186/s12884-024-06409-y)
Supplement: Supplementary file 1 — Supplementary Material 1. [file 12884_2024_6409_MOESM1_ESM.docx]

**Table 2: Newcastle Ottawa quality assessment of prevalence of APD and associated factors**

| **Included Studies** | **Assessment criteria** | | | | | | | |  |
| --- | --- | --- | --- | --- | --- | --- | --- | --- | --- |
|  | Representativeness of the sample | Sample size | Non-respondents | Ascertainment of the exposure (risk factor): | The subjects in different outcome groups are comparable, based on the study design or analysis. Confounding factors are controlled | Assessment of the outcome: | Statistical test | total | Remark |
| Birhane G. et al | 1 | 1 | 1 | 1 | 1 | 2 | 1 | 8 | Good |
| Dibaba et al. | 1 | 1 | 1 | 2 | 1 | 2 | 1 | 9 | Good |
| Tilahun B. et al | 1 | 1 | 1 | 1 | 1 | 2 | 1 | 8 | Good |
| Edao T. et al | 1 | 1 | 1 | 2 | 1 | 2 | 1 | 9 | Good |
| Bitew et al. | 1 | 1 | 1 | 1 | 1 | 2 | 1 | 8 | Good |
| Ayele et al. | 1 | 1 | 1 | 1 | 1 | 2 | 1 | 8 | Good |
| Biratu and Haile | 1 | 1 | 1 | 2 | 1 | 2 | 1 | 9 | Good |
| Bisetegn et al. | 1 | 1 | 1 | 2 | 1 | 2 | 1 | 9 | Good |
| Kasim et al. | 1 | 1 | 1 | 2 | 1 | 2 | 1 | 9 | Good |
| Bekem et al. | 1 | 1 | 1 | 2 | 1 | 2 | 1 | 9 | Good |
| Tamiru et Al | 1 | 1 | 1 | 1 | 1 | 2 | 1 | 8 | Good |
| Tarafa et al. | 1 | 1 | 1 | 2 | 1 | 2 | 1 | 9 | Good |
| Borie et al. | 1 | 1 | 1 | 1 | 1 | 2 | 1 | 8 | Good |
| Beketie et al. | 1 | 1 | 1 | 2 | 1 | 2 | 1 | 9 | Good |
| Abebe et al. | 1 | 1 | 1 | 1 | 1 | 2 | 1 | 8 | Good |
| Beyene et al. | 1 | 1 | 1 | 1 | 1 | 2 | 1 | 8 | Good |
| Shitu Ayen et al | 1 | 1 | 1 | 2 | 1 | 2 | 1 | 9 | Good |
| Yonas and Liyew | 1 | 1 | 1 | 2 | 1 | 2 | 1 | 9 | Good |
